# Nationwide Cross-Sectional Analysis of Mortality Trends in Patients with Sarcoidosis and Non-Ischemic Cardiovascular Disease—The Impact of Gender, Ethnicity, Geographical Location, and COVID-19 Pandemic

**DOI:** 10.3390/jcm13237463

**Published:** 2024-12-08

**Authors:** Raheel Ahmed, Mushood Ahmed, Yehya Khlidj, Obaid Ur Rehman, Laith Al-Mukhtar, Noha Abou Khater, Syed Khurram Mustaq Gardezi, Muhammad Rashid, Peter Collins, Hritvik Jain, Kamleshun Ramphul, Mudassar Baig, Anwar Chahal, Vasilis Kouranos, Nitish Behary Paray, Rakesh Sharma

**Affiliations:** 1 Royal Brompton Hospital, Part of Guy’s and St. Thomas’ NHS Foundation Trust, London SW3 6NP, UK; r.ahmed21@imperial.ac.uk (R.A.); peter.collins@imperial.ac.uk (P.C.); v.kouranos@rbht.nhs.uk (V.K.); rakesh.sharma@rbht.nhs.uk (R.S.); 2 National Heart and Lung Institute, Imperial College London, London SW7 2AZ, UK; 3 Department of Medicine, Rawalpindi Medical University, Rawalpindi 46000, Pakistan; mushood07@gmail.com; 4 Faculty of Medicine, University of Algiers 1, Algiers 16000, Algeria; yehya.khlidj@gmail.com; 5 Services Institute of Medical Sciences, Lahore 54000, Pakistan; i.obaidrehman5@gmail.com; 6 Department of Medicine, University Hospitals Plymouth, Plymouth PL6 8DH, UK; 7 Royal Devon University Healthcare NHS Foundation Trust, Exeter EX2 5DW, UK; n.paray1@nhs.net; 8 Sheikh Shakhbout Medical City, Abu Dhabi P.O. Box 11001, United Arab Emirates; nokhater@ssmc.ae (N.A.K.); syed.gardezi@ku.ac.ae (S.K.M.G.); 9 School of Medicine, Keele University, Staffordshire ST5 5BG, UK; m.rashid@keele.ac.uk; 10 All India Institute of Medical Sciences, Jodhpur 342000, India; hritvikjain2001@gmail.com; 11 Independent Researcher, Triolet 215000, Mauritius; 12 Sunderland Royal Hospital, Sunderland SR4 7TP, UK; mudassarbaig@hotmail.com; 13 Wellspan Clinic, York, PA 17403, USA; anwar.chahal@me.com; 14 Mayo Clinic, Rochester, NY 13400, USA

**Keywords:** sarcoidosis, cardiovascular disease, gender, ethnicity, geographic location, COVID-19

## Abstract

**Background and Objectives:** The epidemiological data regarding mortality rates of adults with sarcoidosis and non-ischemic cardiovascular disease (CVD) are limited. A retrospective observational analysis was conducted to identify trends and disparities related to sarcoidosis and non-ischemic cardiovascular disease mortality among the adult US population from 1999 to 2022. **Methods:** We used the Centers for Disease Control and Prevention (CDC) WONDER database to extract death certificate data for the adult US population (≥25 years). The age-adjusted mortality rates (AAMRs) per 100,000 persons were calculated, and annual percent changes (APCs) were determined using Joinpoint. **Results:** Between 1999 and 2022, 23,642 deaths were identified related to non-ischemic CVD + sarcoidosis. The overall AAMR increased from 0.2 (95% CI, 0.2 to 0.3) in 1999 to 0.5 (95% CI, 0.5 to 0.6) in 2022. Females had a higher AAMR than males (0.6 vs. 0.5). Non-Hispanic (NH) blacks had the highest AAMR, followed by NH whites and Hispanic or Latinos. The southern region had the highest AAMR (0.7: 95% CI, 0.6–0.7), followed by the Midwest (0.6, 95% CI, 0.54–0.669), the Northeast (0.5, 95% CI, 0.5 to 0.6), and the West (0.4; 95% CI, 0.3–0.4). Urban and rural areas had comparable mortality rates (0.5 vs. 0.6). People aged 65+ had the highest AAMRs. **Conclusions:** The overall mortality rates for non-ischemic CVD and sarcoidosis have increased in the US from 1999 to 2022. Females and NH blacks had higher AAMRs, while a minimal variation was observed based on geographical regions. Early diagnosis and prompt management are the keys to reducing the mortality burden of non-ischemic CVD plus sarcoidosis.

## 1. Introduction

A notable increase in sarcoidosis hospitalizations has been witnessed over the past decade with a myriad of coexistent cardiovascular manifestations [1]. Specifically, cardiac sarcoidosis (CS) is being recognized as one of the main causes of mortality in the sarcoidosis population [2,3]. This normally manifests with heart failure, ventricular arrhythmias, cardiac arrest, or high-grade atrioventricular blocks [4]. Expectedly, these presentations are considerably higher among sarcoidosis patients as compared to matched patients without sarcoidosis [5]. Chronic steroid treatment, commonly administered to sarcoidosis patients, has been associated with adverse control of blood pressure, diabetes, obesity, and hyperlipidemia, further exacerbating cardiovascular disease (CVD) risk [6]. This mutual relationship between CVD and sarcoidosis, with one aggravating the other, calls for an in-depth evaluation of the mortality outcomes among patients with sarcoidosis and concomitant non-ischemic CVD.

In the last couple of decades, with the advent of advanced imaging modalities and multidisciplinary team management, the diagnosis and management of systemic sarcoidosis in general and cardiac sarcoidosis in particular has noticed a paradigm shift [7,8,9]. Possibly reflecting heightened clinical suspicion among physicians and wider availability of diagnostic tools, a recent longitudinal analysis revealed that the mortality attributed to CVD in patients with sarcoidosis has risen from 1999 to 2020 [10]. The results of this study were based on a relatively small cohort size secondary to reporting only cardiovascular mortality in sarcoidosis patients. There remains a knowledge gap on whether all-cause mortality in patients with concomitant sarcoidosis and non-ischemic cardiovascular disease follows a similar course, and this study aims to fill this void.

## 2. Methods

### 2.1. Study Population

The National Center for Health Statistics (NCHS) data provided via the Centers for Disease Control and Prevention Wide-Ranging Online Data for Epidemiologic Research (CDC WONDER) database was used to assess the CVD + sarcoidosis mortality rates for adults aged > 25 years from 1999 to 2022. Similar age limits have been used in prior research [11]. The Multiple Cause of Death Public Use Record and International Classification of Diseases, 10th Revision (ICD-10) codes and ICD-10 I00-I99 for CVD, except ischemic heart diseases (I20–I25), sarcoidosis (D86), and COVID-19 (U07.1), were used to extract data. These codes have been used by other researchers in the past [10]. The data were extracted from all death certificates that listed CVD + sarcoidosis as an underlying or contributing cause of death. To analyze the mortality burden of the COVID-19 pandemic, a sensitivity analysis was conducted to assess COVID-19 and sarcoidosis-related deaths from 2020 to 2022. Approval from an institutional review board (IRB) was not required as the study used anonymous data made publicly available. The STROBE guidelines were followed to conduct the study [12].

### 2.2. Data Extraction

The data were extracted for a range of demographic variables that included sex, race/ethnicity, geographical distribution, age groups, and urban–rural classification. Individuals were categorized into the following racial/ethnic groups: Hispanics or Latinos, non-Hispanic (NH) whites, NH black or African American, and NH others (NH Asian or Pacific Islander, Hawaiians, NH American Indian, or Alaska Native, etc.). The age groups included individuals aged 25 to 44 years, 45 to 64 years, and 65+ years. The data were extracted for mortality rates recorded in each state and census region (Northeast, Midwest, South, and West). The urban–rural classification was based on rural, i.e., micropolitan, noncore regions, or urban, i.e., large central metro, large fringe metro, medium metro, small metro regions [13].

### 2.3. Statistical Analysis

The crude mortality rates (CMRs) and age-adjusted mortality rates (AAMRs) for CVD + sarcoidosis were retrieved from the CDC WONDER database. AAMR accounts for differences in the age distribution of the population, enabling data comparison. It was calculated using the direct method of adjustment with the 2000 standard population [14]. The Joinpoint Regression Program (Joinpoint version 5.1.0, National Cancer Institute) was used by researchers to analyze age-adjusted mortality trends from 1999 to 2022 [15]. The program employs serial permutation tests to assess repeated time trends and identifies the inflection point where mortality rate change is statistically significantly different. It then calculates the average annual percent change (APC) for each period in the age-adjusted mortality rate (AAMR), along with the corresponding 95% confidence intervals (CIs). The APC/AAPC/Tau confidence interval was determined using the empirical quartile method. An APC estimate was noted as indicating an increase or decrease if the slope of the trend was significantly different from zero; otherwise, the trend was considered stable. A statistically significant trend change was defined by a *p* value of less than 0.05.

## 3. Results

A total of 23,642 deaths were identified between 1999 and 2022, attributed to non-ischemic CVD + sarcoidosis.

### 3.1. Annual Trends for CVD and Sarcoidosis-Related AAMR

The AAMR for CVD and sarcoidosis-related deaths in adults was 0.292 (95% CI, 0.266 to 0.317) in 1999 and increased to 0.594 (95% CI, 0.565 to 0.624) in 2022. The AAMR exhibited a consistent rise from 1999 to 2018 (APC: 1.6577; 95% CI, 0.7521 to 2.197; *p*: 0.001), followed by a significant increase from 2018 to 2022 (APC: 7.8438; 95% CI, 4.1007 to 15.5501; *p* < 0.001, Table S1).

### 3.2. CVD and Sarcoidosis-Related AAMR Stratified by Sex

**Figure 1:**
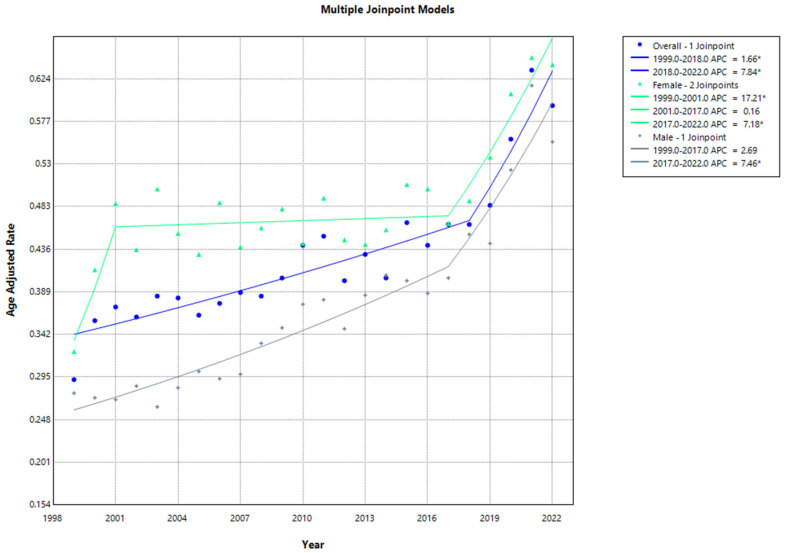
Joinpoint analysis of age-adjusted mortality rates for cardiovascular disease and sarcoidosis stratified by overall population and sex (1999–2022). * Indicates a statistically significant change in AAMR trends.

The AAMR for females consistently exceeded that of males over the study period. The AAMR for females stood at 0.323 (95% CI, 0.287 to 0.359) in 1999, increasing to 0.486 (95% CI, 0.442 to 0.580) in 2001 (APC: 17.2060; 95% CI, 1.5256 to 37.3827; *p*: 0.01), followed by a decline to 0.464 (95% CI, 0.427 to 0.502) in 2017 (APC: 0.1616; 95% CI, −7.9632 to 0.7844; *p* > 0.05). AAMR notably increased to 0.6399 (95% CI, 0.598 to 0.681) in 2022 (APC: 7.1832; 95% CI, 2.994 to 17.0697; *p* < 0.05). Similarly, the AAMR for males in 1999 was 0.277 (95% CI, 0.238 to 0.315), progressively rising to 0.404 (95% CI, 0.368 to 0.441) in 2017 (APC: 2.6854; 95% CI, −0.1133 to 3.4762; *p* > 0.05), followed by an increase to 0.554 (95% CI, 0.511 to 0.598) in 2022 (APC: 7.4634; 95% CI, 4.0588 to 15.6965; *p* < 0.000001) (Tables S2 and S3, Figure 1).

### 3.3. CVD and Sarcoidosis-Related AAMR Stratified by Race/Ethnicity

The highest AAMRs were observed among NH black or African American group, followed by NH whites. The NH black or African American group exhibited an AAMR of 1.633 in 1999, which increased to 2.06 in 2017 (APC: 0.2628; 95% CI, −0.9015 to 0.9155; *p* = 0.586683), followed by a further increase to 2.563 in 2022 (APC: 6.0585; 95% CI, 2.7913 to 14.6757; *p* < 0.000001).

**Figure 2:**
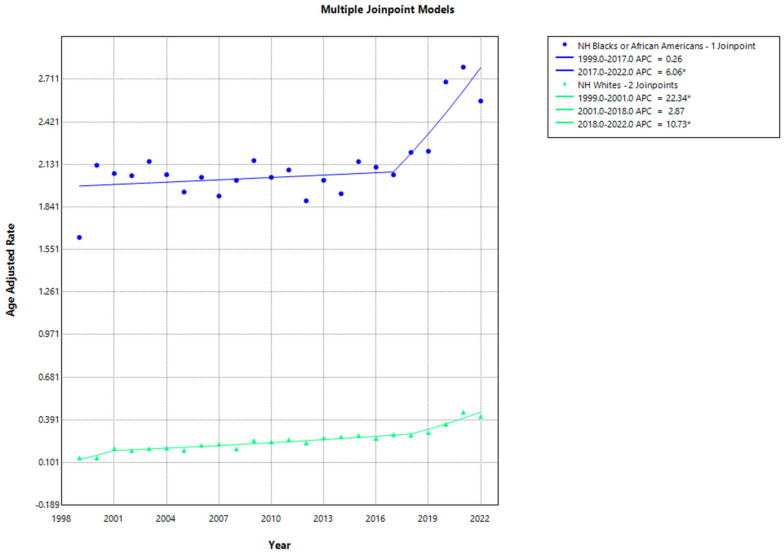
Joinpoint analysis of age-adjusted mortality rates cardiovascular disease and sarcoidosis stratified by race from 1999–2022. * Indicates a statistically significant change in AAMR trends.

The mortality rate for NH white group also demonstrated an increasing trend from 0.132 to 0.196 from 1999 to 2001 (APC: 22.3406; 95% CI, 4.4622 to 39.741; *p* < 0.000001), with a rise to 0.286 in the mortality rate from 2001 to 2018 (APC: 2.8652; 95% CI, −3.0608 to 3.5123; *p* = 0.119176), followed by an increase to 0.414 from 2018 till 2022 (APC: 10.7260; 95% CI, 5.725 to 20.0315; *p* < 0.000001). Between 2009 and 2022, the AAMR for Hispanics or Latinos increased from 0.128 to 0.181 (Table S4, Figure 2).

### 3.4. CVD and Sarcoidosis-Related AAMR Stratified by Geographic Region

State. Between 1999 and 2020, the highest AAMR of 1.799 was displayed in the District of Columbia. South Carolina exhibited the second-highest AAMR of 0.938, while New Mexico reflected the lowest AAMR at 0.106. The AAMR for other states fell between Maryland (0.853) and New Mexico. Subsequently, a discernible increase in AAMR was documented during the 2021–2022 period for several states. States that ranked in the upper 90th percentile for CVD and sarcoidosis-related mortalities in 2021–2022 included the District of Columbia, South Carolina, Maryland, Minnesota, and Mississippi. Conversely, states within the lower 10th percentile in the same period included Arizona, California, Missouri, and Florida (Tables S5 and S6).

**Figure 3:**
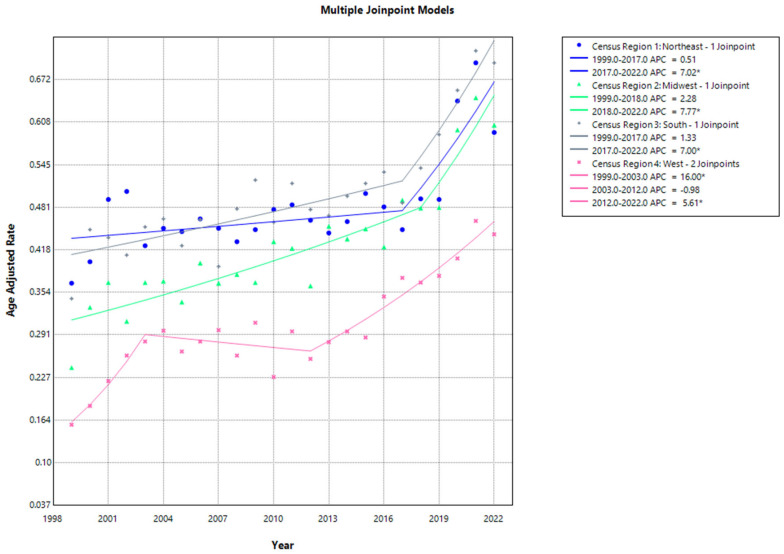
Joinpoint analysis of age-adjusted mortality rates for cardiovascular disease and sarcoidosis stratified by census region (1999–2022). * Indicates a statistically significant change in AAMR trends.

Census Region. In 1999, the AAMR for the Northeast region was 0.368 (95% CI, 0.306 to 0.431), which experienced a steady increase to 0.448 (95% CI, 0.385 to 0.511) by 2017 (APC: 0.51; 95% CI, −1.5266 to 1.3125; *p* = 0.433513), followed by a steep increase to 0.593 (95% CI, 0.524 to 0.662) till 2022 (APC: 7.02; 95% CI, 2.8297 to 17.8649; *p* = 0.000001). The Midwest region had an AAMR of 0.242 (95% CI, 0.277 to 0.387) in 1999, which gradually climbed to 0.48 (95% CI, 0.421 to 0.538) in 2018 (APC: 2.28; 95% CI, −3.4439 to 12.7091; *p* = 0.143971) and increased to 0.604 (95% CI, 0.54 to 0.669) in 2022 (APC: 7.77; 95% CI, 2.7069 to 18.0707; *p* = 0.013597). The AAMR for the South region showed a stable rise from 0.345 to 0.488 between 1999 and 2017 (APC: 1.33; 95% CI, −0.2185 to 2.0383; *p* = 0.063987), followed by a notable surge, resulting in an AAMR of 0.656 by 2022 (APC: 7.0; 95% CI, 3.5452 to 16.0071; *p* < 0.000001). Similarly, the West region’s AAMR was 0.157 in 1999, which increased steadily to 0.281 in 2003 (APC: 16.0; 95% CI, 8.7507 to 31.0257; *p* < 0.000001) and declined to 0.255 by 2012 (APC: −0.98; 95% CI, −7.0707 to 0.9569; *p* = 0.280344), followed by a notable increase to 0.441 by 2022 (APC: 5.61; 95% CI, 4.2938 to 7.9614; *p* = 0.0004). Overall, during the period of study spanning from 1999 to 2022, it was observed that the Northeast region exhibited the highest AAMR at 0.697. Between 1999 and 2022, the West region consistently demonstrated the lowest AAMR, registering values between 0.157 and 0.461 (Table S7, Figure 3).

**Figure 4:**
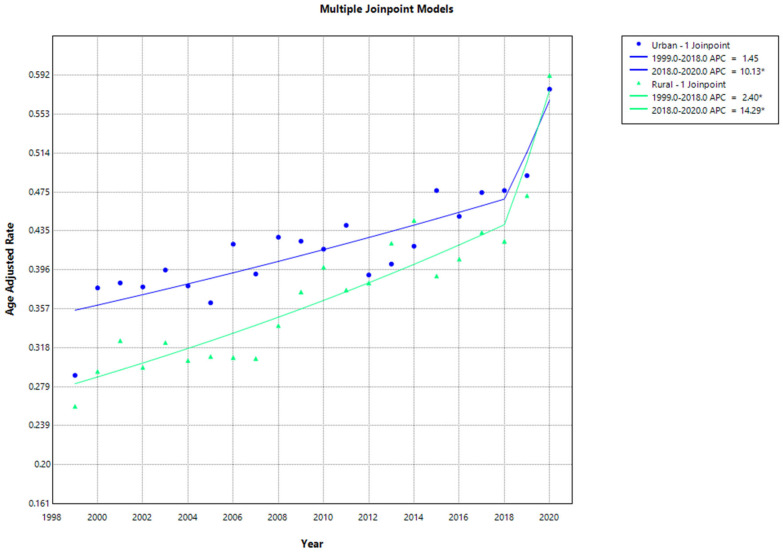
Joinpoint analysis of age-adjusted mortality rates for cardiovascular disease and sarcoidosis stratified by urbanization (1999–2020). * Indicates a statistically significant change in AAMR trends.

Urban–Rural. From 1999 to 2020, non-metropolitan areas and metropolitan areas displayed comparable CVD and sarcoidosis-related AAMR. Specifically, the AAMR in metropolitan areas steadily increased from 0.29 (95% CI, 0.262 to 0.317) in 1999 to 0.476 (95% CI, 0.447 to 0.505) in 2018 (APC: 1.45; 95% CI, −0.8979 to 2.6139; *p* = 0.078784), followed by a steep incline to 0.578 (95% CI, 0.546 to 0.610) in 2020 (APC = 10.13; 95% CI, 1.8347 to 15.1317; *p* < 0.000001). Similarly, the AAMR for non-metropolitan areas consistently increased from 0.259 (95% CI, 0.203 to 0.326) in 1999 to 0.425 (95% CI, 0.357 to 0.492) in 2018 (APC: 2.40; 95% CI, 0.1528 to 3.2972; *p* = 0.04879), and a significant rise to 0.592 (95% CI, 0.516 to 0.668) was observed in 2020 (APC: 14.29; 95% CI, 3.1005 to 20.5045; *p* < 0.000001). Data for AAMR were unavailable for 2021–2022, based on CDC provisional data (Table S8, Figure 4).

### 3.5. CVD and Sarcoidosis-Related AAMR Stratified by Age Groups

**Figure 5:**
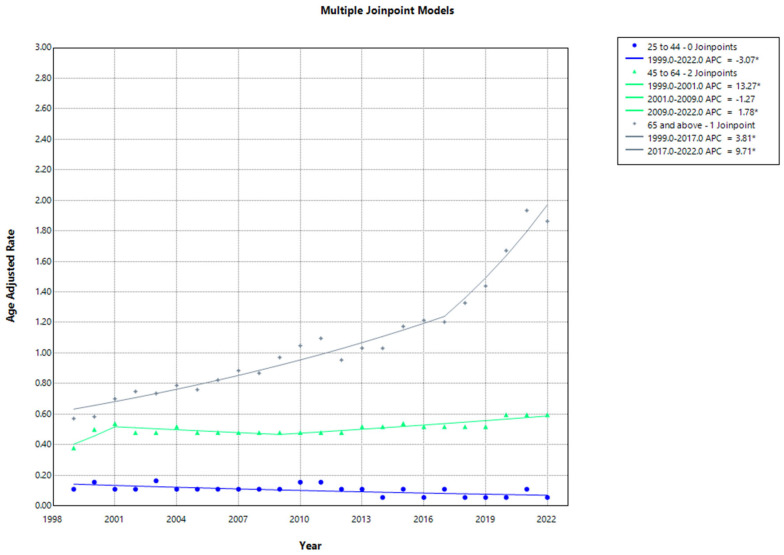
Joinpoint analysis of age-adjusted mortality rates for stratified by age groups (1999–2022). * Indicates a statistically significant change in AAMR trends.

In the analysis stratified by age groups, the highest AAMR was observed in the 65 and above age categories, followed by the 45–64 age group. Conversely, the 25–44 age group displayed the lowest AAMR. CVD and sarcoidosis-related mortality in both the 25–44 years of age group decreased from 1999 to 2022 (APC: −3.07; 95% CI, −5.4146 to −0.9892; *p* = 0.004799). Moreover, the AAMRs for the 45–64 age segment consistently increased from 1999 to 2001 (APC: 13.27; 95% CI, 1.73 to 26.1313; *p* = 0.019596), followed by a downsurge until 2009 (APC: −1.27; 95% CI, −6.3744 to 0.4271; *p* = 0.104379). A relatively moderate increase in the AAMR was observed between 2009 and 2022 for the 45-to-64-years-of-age group (APC: 1.78; 95% CI, 0.9616 to 4.2321; *p* = 0.034393). As for the 65+ age group, the AAMR demonstrated a steady incline from 1999 to 2017 (APC: 3.81; 95% CI, 2.6167 to 4.5294; *p* = 0.004799), with a marked increase observed from 2017 to 2022 (APC: 9.71; 95% CI, 6.7781 to 16.3435; *p* < 0.000001) (Table S9, Figure 5).

### 3.6. COVID-19 and Sarcoidosis-Related AAMR from 2020 to 2022

A total of 1185 deaths were recorded in the US from 2020 to 2022 related to COVID-19 and sarcoidosis. The AAMRs peaked in 2021 at 0.16 (0.15–0.18), followed by a decline in 2022 (AAMR: 0.09, Table S10).

## 4. Discussion

This nationwide cross-sectional analysis provides critical insights into the disparities and trends in all-cause mortality of patients with sarcoidosis and non-ischemic cardiovascular disease. The mortality rates in this cohort of patients have doubled from 1999 to 2022, characterized by a gradual and steady rise up to 2018, followed by a sudden and steep increase from 2018 to 2022. Patients with age > 65 years, female sex, and black ancestry exhibited the highest AAMR. These factors likely contributed to regional variations in AAMR, whereby the District of Columbia and the Northeast region showed the highest rates.

### 4.1. General Rise till 2018

Cardiac involvement in systemic sarcoidosis is increasingly recognized despite remaining a relatively rare condition. The increase in the rates of diagnosis of CS—and, by extension, in the rates of mortality attributed to this condition—can be linked to a number of factors. Firstly, CS was historically underdiagnosed due to its subtle or non-specific presentations, such as arrhythmias or unexplained heart failure [2]. In the absence or limited availability of diagnostic tools, patients with early stages of cardiac involvement went undiagnosed, and the majority of cases of cardiac involvement in systemic sarcoidosis were found post mortem [16]. Secondly, clinical societies, such as the Heart Rhythm Society and the Japanese Circulation Society, addressed the discrepancies in diagnostic approach by developing structured guidelines in the 2010s, based on expert consensus [17,18]. These guidelines delineated a proactive approach to screening in high-risk patients with systemic sarcoidosis, thereby contributing to the detection of CS prior to the onset of serious cardiac manifestations. Finally, the advent of advanced imaging techniques in the 2000s, particularly cardiac magnetic resonance imaging (cMRI) and positron emission tomography (PET) scans, provided a more reliable way to detect cardiac inflammation and scarring, even in individuals with minimal to no symptoms [19]. Studies assessing the use of cMRI and PET in the suspected or general sarcoidosis population have also highlighted the prognostic value of these investigations [20,21,22]. Exemplifying the impact of these factors, a nationwide cohort study in Finland by Pöyhönen et al. found that the rate of diagnosis of CS increased by more than 100-fold between 1988 and 2019 [9]. Rather than solely reflecting a true increase in the incidence of CS, the authors proposed that the rise in diagnostic rates likely stemmed from a combination of a guideline-directed approach to evaluating cardiac involvement in systemic sarcoidosis patients, greater availability of diagnostic resources, and heightened awareness among clinicians. This correlated with a generally less aggressive phenotype and better prognosis, potentially indicating that CS was being detected at an earlier stage [9].

### 4.2. Rapid Rise from 2018 to 2022

The present study yielded a surprising observation of a roughly five-fold increase in annualized mortality rates in patients with systemic sarcoidosis and concurrent non-ischemic CVD from 2018 to 2022 compared to preceding years. The authors posit that this sudden surge could reflect the direct and indirect effects of the COVID-19 pandemic. One of the major impacts of the pandemic was the delay in diagnoses due to interruptions in routine care caused by strain on the healthcare system and reallocation of resources to hospitalized patients with COVID-19 [23]. Elective outpatient follow-ups, essential for monitoring disease activity and treatment efficacy, were often interrupted due to clinic closures or restrictions. As a result, the early phase of the pandemic saw a significant number of cancellations and a reduction in service provision [24]. Novel triage recommendations and risk-based scoring assessments [25,26] were implemented to mitigate the impact on patients perceived to be at highest risk. While the intended benefit was to facilitate the management of urgent cases, these measures caused a considerable decrease in cardiovascular investigations and interventions being performed [27]. Another critical aspect of the care of patients with sarcoidosis during the pandemic was the dilemma surrounding the use of immunosuppressive therapy. The risks of ongoing inflammation and disease progression had to be carefully weighed against the potentially higher risk of severe COVID-19 infection. This balancing act was greatly complicated by the limited amount of literature initially available to guide physicians given the unprecedented nature of the pandemic [28]. Furthermore, medication non-adherence became a common problem among patients, likely as a self-management strategy in those concerned about the perceived risks of more severe COVID-19 clinical course while on immunosuppressive regimens [29]. Finally, small case series have demonstrated how COVID-19 can overlap with CS to precipitate myocardial inflammation and increase arrhythmogenicity [30,31]. When combined, these factors could underlie the steep increase in mortality rates seen between 2018 and 2022.

### 4.3. Gender Disparity

Female patients with sarcoidosis and non-ischemic CVD were noted have a higher AAMR than males. The causes of sex differences in the prognosis of patients with systemic sarcoidosis are challenging to study and the exact reasons for these observed trends in mortality rates remain unelucidated. It is more than likely that a complex interplay of factors linked to comorbidities, demographics, access to healthcare services, and systemic biases causing diagnostic delays, among others, underlie these differences. Evidence is also emerging that the phenotypic expression of CS varies based on biological sex, with males showing more arrhythmic variants and females showing predominantly heart failure variants [32]. Our observations mirror the findings of few previous studies [10,33,34], contributing to the hypothesis-generating literature and underscoring the importance of further prospective confirmatory research.

### 4.4. Ethnic/Race Disparity

African Americans are more likely to die from sarcoidosis [35]. There are several potential explanations for this, including genetic predisposition. An example of a gene implicated is the HLA-DQB1*0602 allele, which was found to be linked with radiographic progression of disease in US black families [36]. In another study, for the same sarcoidosis stage, a higher granuloma density was observed among African Americans with respect to other races [37], which could be attributed to a more prominent interaction between pro-inflammatory genes and exogenous (e.g., environmental) triggers in people of black ancestry. Indeed, black US individuals were demonstrated to display significantly higher exposure to PM_2.5_ and NO_2_ compared with non-Hispanic white individuals, and this was associated with a worse DLCO % predicted value in sarcoid lungs in another study [38]. Biased healthcare delivery driven by lower socioeconomic status is also an important potential contributor to this race-based disparity [39]. Black participants with sarcoidosis have also reported lower medication adherence than white participants [40].

### 4.5. State and Region Disparity

Geographic characterization plays a key role in understanding the epidemiology of autoimmune diseases. We observed a latitude gradient of mortality in patients with sarcoidosis and CVD where the states of District of Columbia and South Carolina displayed the highest and second-highest AAMR, respectively. The two states harbor significant proportions of black ancestry population [41], which would result in higher vulnerability to deaths by sarcoidosis and CVD. Residents of the District of Columbia have the highest personal incomes per capita [42]. Additionally, the number of centers providing cardiac MRI per million persons in the US was shown to be the highest in the District of Columbia [43]. Both of these factors would be suggestive of a higher accessibility to MRI and PET scans locally, notably resulting in more diagnosed cases and deaths from cardiac sarcoidosis/sarcoidosis with cardiovascular comorbidities. At the same time, in the District of Columbia the poverty rate is 14.0% [44], and the local unemployment rate is the highest in the country [45]. Therefore, the economically disadvantaged populations in this state could be at additional risk of a sarcoidosis-related poor prognosis as the latter is strongly correlated with low financial status [46]. These same reasons could explain the observed increased mortality in the Northeast region, where the District of Columbia and South Carolina states are located.

### 4.6. No Disparity in Urban vs. Rural Groups

Paradoxically, our study revealed no significant disparity in AAMR according to urbanization status. These results differ from those of Tan et al.’s study in which CVD mortality with comorbid sarcoidosis was more common in the urban regions [10]. This disaccord could be driven by the initially mentioned differences in the methodology. Importantly, in the USA from 1999 to 2020, CVD-related mortality was less prominent in urban areas compared to rural areas [47]. However, patients living in urban areas likely benefited from higher cardiac MRI/PET scan centers, leading to an increase in diagnosis and apparent mortality. Overall, these two factors may have neutralized the all-cause mortality in the urban subpopulation.

### 4.7. Age Disparity

Not surprisingly, we found significant differences in the AAMR across age groups. Elderly people with sarcoidosis and non-ischemic CVD have a significantly higher likelihood of mortality as compared to their younger counterparts [10,48]. The age category of patients aged ≥ 65 years would be the most vulnerable to sarcoidosis burden mainly due to greater comorbidities. They would also be at higher risk of developing mortality from CVD and advanced sarcoidosis-related complications (due to a longer disease course), including the risks of chronic corticosteroid therapy [49]. Interestingly, the mortality was lower in 25–44-year-old age groups. This is in line with the findings of Swigris et al., who observed a decline in mortality rates in patients aged 25–34 years and an incline in those 45–84 years during the period of 1988 to 2007 [33]. Such observations could be reflective of more focused healthcare toward young groups with sarcoidosis and concomitant CVD but also improvements in the management of early-onset forms of cardiac/non-cardiac sarcoidosis.

### 4.8. Strengths and Limitations

The strength of the present analysis lies in the sizeable number of cases studied. In comparison to Tan et al., there were over seven times the number of reported deaths, which enables sufficient statistical power to identify disparities. By adding the 2021–2022 data, this study also demonstrates the impact of the COVID-19 pandemic, in which a surge in mortality was observed.

Whilst our study provides valuable insights, several limitations must be acknowledged. First, the reliance on death certificate data may introduce potential inaccuracies due to variations in coding practices and the possibility of underreporting sarcoidosis as a cause of death. The CDC WONDER database does not have the granularity in data such as details on baseline demographics, investigation findings, or treatment regimens, including cardiac devices data that individual patients had [50]. Furthermore, as data for 2021–2022 remain provisional for urban–rural data, the impact of emerging trends following the COVID-19 pandemic may not be fully captured. Lastly, the observational nature of our study cannot establish causal relationships, necessitating further research to explore the underlying factors influencing the observed trends.

## 5. Conclusions

Mortality rates in patients with concurrent CVD and sarcoidosis have risen over the last two decades with a remarkable surge noted in the period of the COVID-19 pandemic. Factors associated with worse mortality include sociodemographic factors such as female sex, black ethnicity, and advanced age. These findings add to the growing literature on ‘big data’ analyses in sarcoidosis and underscore the need for prospective studies to help inform personalized preventative strategies aimed at reducing the burden of morbidity and mortality in this highly diverse cohort of patients.

## Data Availability

The generated data can be found in https://wonder.cdc.gov/.

## Supplementary Materials

The following supporting information can be downloaded at https://www.mdpi.com/article/10.3390/jcm13237463/s1, Table S1: Cardiovascular Disease and Sarcoidosis-deaths, Stratified by Sex and Race in the United States, 1999 to 2022. Table S2: Annual percent change (APC) of Cardiovascular Disease and Sarcoidosis Related Age-Adjusted Mortality Rates per 100,000 in the United States, 1999 to 2022. Table S3: S3: Overall and Sex-Stratified Cardiovascular Disease and Sarcoidosis Related Age-Adjusted Mortality Rates per 100,000 in the United States, 1999 to 2022. Table S4: Cardiovascular Disease and Sarcoidosis-Related Age-Adjusted Mortality Rates per 100,000, Stratified by Race in the United States, 1999 to 2022. Table S5: Cardiovascular Disease and Sarcoidosis-Related Age-Adjusted Mortality Rates per 100,000, Stratified by States in the United States, 1999 to 2020. Table S6: Cardiovascular Disease and Sarcoidosis-Related Age-Adjusted Mortality Rates per 100,000, Stratified by States in the United States, 2021 to 2022. Table S7: Cardiovascular Disease and Sarcoidosis-Related Age-Adjusted Mortality Rates per 100,000, Stratified by Census Region in the United States, 1999 to 2022. Table S8: Cardiovascular Disease and Sarcoidosis Related Age-Adjusted Mortality Rates per 100,000 in United States stratified by Urban-Rural Classification, 1999-2020. Table S9: Cardiovascular Disease and Sarcoidosis-Related Age-Adjusted Mortality Rates (AAMR) per 100,000 individuals Stratified by Ten Year Age Groups in the United States, 1999 to 2022. Table S10: Sarcoidosis and COVID-19-related Age-Adjusted Mortality Rates per 100,000 in the United States from 2020 to 2022.
