# Supplementary material for: Nationwide Cross-Sectional Analysis of Mortality Trends in Patients with Sarcoidosis and Non-Ischemic Cardiovascular Disease—The Impact of Gender, Ethnicity, Geographical Location, and COVID-19 Pandemic
Source: J Clin Med. 2024 Dec 8;13(23):7463. doi: 10.3390/jcm13237463 (PMC11642065; doi:10.3390/jcm13237463)
Supplement: Supplementary file 1 [file jcm-3331429-supplementary.pdf › jcm-3331429-supplementary.pdf]

Supplementary appendix

Journal of Clinical Medicine

Nationwide Cross-sectional Analysis of Mortality Trends in Patients with Sarcoidosis and Non-ischemic Cardiovascular Disease - The Impact of Gender, Ethnicity, Geographical Location and COVID-19 Pandemic

Raheel Ahmed, Mushood Ahmed, Yehya Khldij, Obaid Ur Rehman, Laith Al-Mukhtar, Noha Abou Khater, Syed Khurram Mustaq Gardezi, Muhammad Rashid, Peter Collins, Hritvik Jain, Kamleshun Ramphul, Mudassar Baig, Anwar Chahal, Vasilis Kouranos, Nitish Behary Paray, Rakesh Sharma

Corresponding author:

Laith Al-Mukhtar

Royal Devon University Healthcare NHS Foundation Trust

laith.al-mukhtar@nhs.net

+447545307162

Table S1. Cardiovascular Disease and Sarcoidosis-deaths, Stratified by Sex and Race in the United States, 1999 to 2022

	Deaths					
**Year**	**Female**	**Male**	**NH White**	**NH Black or African American**	**NH Asian or Pacific Islander**	**Hispanic or Latino**
**1999**	316	205	205	299	Suppressed	10
**2000**	418	227	226	399	Suppressed	14
**2001**	479	226	285	398	Suppressed	13
**2002**	445	255	278	402	Suppressed	13
**2003**	485	245	276	427	Suppressed	19
**2004**	484	279	319	420	Suppressed	19
**2005**	469	260	287	413	Suppressed	24
**2006**	533	276	341	440	Suppressed	22
**2007**	495	286	342	412	Suppressed	21
**2008**	520	315	351	457	10	15
**2009**	554	354	383	490	Suppressed	22
**2010**	530	376	392	475	Suppressed	27
**2011**	591	359	415	498	Suppressed	27
**2012**	552	341	401	456	Suppressed	24
**2013**	582	410	451	509	11	20
**2014**	610	413	476	497	Suppressed	41
**2015**	634	446	485	556	Suppressed	23
**2016**	670	441	496	563	17	33
**2017**	640	494	522	562	18	29
**2018**	698	542	564	614	22	38
**2019**	761	523	609	625	11	38
**2020**	910	652	720	772	16	52
**2021**	962	747	815	802	31	61
**2022**	946	686	803	731	37	58

NH, non-Hispanic.

Table S2. Annual percent change (APC) of Cardiovascular Disease and Sarcoidosis Related Age-Adjusted Mortality Rates per 100,000 in the United States, 1999 to 2022

Year Interval	APC (95% CI)
**Overall**	
1999-2018	1.6577* (0.7521-2.1927)
2018-2022	7.8438* (4.1007-15.5501)
**Male**	
1999-2017	2.6854 (-0.1133-3.4762)
2018-2022	7.4634* (4.0588-15.6965)
**Female**	
1999-2001	17.2060* (1.5256-37.3827)
2001-2017	0.1616 (-7.9632-0.7844)
2017-2022	7.1832* (2.994-17.0697)
**NH White**	
1999-2001	22.3406* (4.4622-39.741)
2001-2018	2.8652 (-3.0608-3.5123)
2018-2022	10.7260* (5.725-20.0315)
**NH Black or African American**	
1999-2017	0.2628 (-0.9015-0.9155)
2017-2022	6.0585* (2.7913-14.6757)
**Rural areas**	
1999-2018	2.3961* (0.1528-3.2972)
2018-2020	14.2940* (3.1005-20.5045)
**Urban area**	
1999-2018	1.4503 (-0.8979-2.6139)
2018-2020	10.1316* (1.8347-15.1317)
**Age**	
**25-44 years**	
1999-2022	-3.0651* (-5.4146--0.9892)
**45-64 years**	
1999-2001	13.2709* (1.73-26.1313)
2001-2009	-1.2733 (-6.3744-0.4271)
2009-2022	1.7828* (0.9616-4.2321)
**65+ years**	
1999-2017	3.8073* (2.6171-4.5294)
2017-2022	9.7105* (6.7781-16.3435)

Table S3. Overall and Sex-Stratified Cardiovascular Disease and Sarcoidosis Related Age-Adjusted Mortality Rates per 100,000 in the United States, 1999 to 2022

	Age-Adjusted Rate (95% CI)		
**Year**	**Male**	**Female**	**Overall**
**1999**	0.469 0.46-0.477)	0.323 0.287-0.359)	0.292 (0.266-0.317)
**2000**	0.277 0.238-0.315)	0.413 0.373-0.453)	0.357 (0.329-0.385)
**2001**	0.272 0.236-0.307)	0.486 0.442-0.53)	0.372 (0.344-0.4)
**2002**	0.27 0.234-0.306)	0.435 0.394-0.476)	0.361 (0.334-0.388)
**2003**	0.285 0.25-0.321)	0.502 0.456-0.547)	0.384 (0.355-0.412)
**2004**	0.262 0.228-0.296)	0.453 0.412-0.494)	0.382 (0.355-0.41)
**2005**	0.283 0.248-0.317)	0.43 0.39-0.469)	0.363 (0.336-0.39)
**2006**	0.301 0.264-0.339)	0.487 0.445-0.53)	0.376 (0.349-0.402)
**2007**	0.293 0.258-0.329)	0.438 0.398-0.477)	0.388 (0.36-0.415)
**2008**	0.298 0.262-0.334)	0.459 0.419-0.5)	0.384 (0.357-0.411)
**2009**	0.332 0.294-0.369)	0.48 0.439-0.52)	0.404 (0.378-0.431)
**2010**	0.349 0.312-0.386)	0.442 0.403-0.481)	0.44 (0.411-0.47)
**2011**	0.375 0.336-0.414)	0.492 0.451-0.533)	0.45 (0.42-0.479)
**2012**	0.38 0.34-0.42)	0.446 0.408-0.483)	0.401 (0.374-0.428)
**2013**	0.348 0.31-0.387)	0.441 0.404-0.477)	0.43 (0.402-0.458)
**2014**	0.385 0.346-0.423)	0.457 0.42-0.494)	0.404 (0.379-0.43)
**2015**	0.407 0.366-0.448)	0.507 0.466-0.548)	0.465 (0.436-0.494)
**2016**	0.401 0.363-0.44)	0.502 0.463-0.542)	0.44 (0.414-0.467)
**2017**	0.387 0.349-0.425)	0.464 0.427-0.502)	0.463 (0.435-0.492)
**2018**	0.404 0.368-0.441)	0.489 0.452-0.526)	0.463 (0.436-0.489)
**2019**	0.452 0.412-0.491)	0.537 0.498-0.577)	0.484 (0.457-0.511)
**2020**	0.442 0.403-0.482)	0.607 0.567-0.648)	0.557 (0.528-0.585)
**2021**	0.616 (0.57-0.662)	0.647 (0.605-0.688)	0.633 0.602-0.664)
**2022**	0.554 (0.511-0.598)	0.639 (0.598-0.681)	0.594 (0.565-0.624)

Table S4. Cardiovascular Disease and Sarcoidosis-Related Age-Adjusted Mortality Rates per 100,000, Stratified by Race in the United States, 1999 to 2022

	Age-Adjusted Rate (95% CI)			
**Year**	**NH White**	**NH Black or African American**	**NH Other Races**	**Hispanic or Latino**
**1999**	0.132 (0.113-0.151)	1.633 (1.446-1.821)	Suppressed (Suppressed-Suppressed)	Unreliable (0.024-0.141)
**2000**	0.132 (0.114-0.15)	2.125 (1.913-2.336)	Suppressed (Suppressed-Suppressed)	Unreliable (0.077-0.246)
**2001**	0.196 (0.172-0.22)	2.069 (1.863-2.275)	Suppressed (Suppressed-Suppressed)	Unreliable (0.056-0.191)
**2002**	0.182 (0.159-0.204)	2.054 (1.85-2.258)	Suppressed (Suppressed-Suppressed)	Unreliable (0.05-0.168)
**2003**	0.196 (0.172-0.221)	2.151 (1.945-2.358)	Suppressed (Suppressed-Suppressed)	Unreliable (0.067-0.197)
**2004**	0.2 (0.177-0.224)	2.061 (1.86-2.261)	Suppressed (Suppressed-Suppressed)	Unreliable (0.058-0.187)
**2005**	0.184 (0.162-0.206)	1.943 (1.753-2.133)	Suppressed (Suppressed-Suppressed)	0.143 (0.086-0.224)
**2006**	0.218 (0.193-0.242)	2.043 (1.849-2.238)	Suppressed (Suppressed-Suppressed)	0.157 (0.097-0.24)
**2007**	0.226 (0.201-0.252)	1.915 (1.727-2.104)	Suppressed (Suppressed-Suppressed)	0.116 (0.07-0.181)
**2008**	0.194 (0.173-0.215)	2.022 (1.833-2.212)	Unreliable (0.045-0.204)	Unreliable (0.041-0.146)
**2009**	0.25 (0.224-0.276)	2.157 (1.961-2.353)	Suppressed (Suppressed-Suppressed)	0.128 (0.076-0.203)
**2010**	0.242 (0.217-0.267)	2.043 (1.854-2.232)	Suppressed (Suppressed-Suppressed)	0.141 (0.09-0.21)
**2011**	0.256 (0.23-0.282)	2.093 (1.904-2.281)	Suppressed (Suppressed-Suppressed)	0.145 (0.092-0.217)
**2012**	0.234 (0.211-0.258)	1.883 (1.705-2.061)	Suppressed (Suppressed-Suppressed)	0.129 (0.081-0.195)
**2013**	0.269 (0.242-0.296)	2.023 (1.843-2.204)	Unreliable (0.043-0.163)	0.089 (0.05-0.147)
**2014**	0.275 (0.25-0.301)	1.931 (1.756-2.105)	Suppressed (Suppressed-Suppressed)	0.171 (0.118-0.241)
**2015**	0.285 (0.258-0.312)	2.15 (1.966-2.333)	Suppressed (Suppressed-Suppressed)	0.115 (0.072-0.173)
**2016**	0.266 (0.241-0.29)	2.112 (1.932-2.292)	Unreliable (0.082-0.226)	0.136 (0.089-0.2)
**2017**	0.291 (0.263-0.318)	2.06 (1.884-2.235)	Unreliable (0.078-0.214)	0.121 (0.079-0.177)
**2018**	0.286 (0.26-0.313)	2.213 (2.034-2.393)	0.156 (0.094-0.244)	0.144 (0.099-0.203)
**2019**	0.304 (0.278-0.329)	2.221 (2.043-2.4)	Unreliable (0.036-0.138)	0.122 (0.081-0.176)
**2020**	0.362 (0.335-0.39)	2.693 (2.498-2.888)	Unreliable (0.057-0.176)	0.19 (0.14-0.251)
**2021**	0.444 (0.412-0.476)	2.794 (2.594-2.993)	0.152 (0.099-0.222)	0.221 (0.167-0.286)
**2022**	0.414 (0.384-0.445)	2.563 (2.372-2.754)	0.198 (0.138-0.275)	0.181 (0.134-0.241)

NH = non-Hispanic. Unreliable: Rates are marked as "unreliable" when the death count is less than 20. (This is done to protect the personal privacy of individuals)

Table S5. Cardiovascular Disease and Sarcoidosis-Related Age-Adjusted Mortality Rates per 100,000, Stratified by States in the United States, 1999 to 2020

State	Age-Adjusted Rate (95%CI)
Alabama	0.637 (0.578-0.696)
Alaska	0.411 (0.278-0.587)
Arizona	0.146 (0.123-0.169)
Arkansas	0.372 (0.313-0.431)
California	0.295 (0.28-0.31)
Colorado	0.369 (0.323-0.416)
Connecticut	0.414 (0.36-0.467)
Delaware	0.571 (0.453-0.711)
District of Columbia	1.799 (1.512-2.087)
Florida	0.305 (0.285-0.324)
Georgia	0.625 (0.583-0.668)
Hawaii	Unreliable (0.034-0.108)
Idaho	0.269 (0.204-0.349)
Illinois	0.345 (0.32-0.371)
Indiana	0.375 (0.338-0.413)
Iowa	0.296 (0.247-0.346)
Kansas	0.332 (0.277-0.386)
Kentucky	0.259 (0.219-0.298)
Louisiana	0.424 (0.375-0.473)
Maine	0.405 (0.325-0.499)
Maryland	0.853 (0.791-0.915)
Massachusetts	0.307 (0.274-0.34)
Michigan	0.527 (0.49-0.564)
Minnesota	0.389 (0.346-0.432)
Mississippi	0.733 (0.651-0.816)
Missouri	0.328 (0.291-0.364)
Montana	0.325 (0.243-0.425)
Nebraska	0.358 (0.287-0.429)
Nevada	0.219 (0.174-0.272)
New Hampshire	0.263 (0.201-0.338)
New Jersey	0.48 (0.444-0.516)
New Mexico	0.106 (0.073-0.148)
New York	0.567 (0.54-0.594)
North Carolina	0.732 (0.688-0.776)
North Dakota	0.283 (0.188-0.409)
Ohio	0.515 (0.482-0.548)
Oklahoma	0.363 (0.314-0.412)
Oregon	0.333 (0.285-0.381)
Pennsylvania	0.449 (0.42-0.478)
Rhode Island	0.431 (0.34-0.54)
South Carolina	0.938 (0.865-1.011)
South Dakota	0.278 (0.196-0.384)
Tennessee	0.448 (0.405-0.491)
Texas	0.318 (0.298-0.337)
Utah	0.342 (0.275-0.409)
Vermont	0.568 (0.426-0.741)
Virginia	0.595 (0.551-0.64)
Washington	0.42 (0.379-0.461)
West Virginia	0.329 (0.267-0.392)

Wisconsin	0.417 (0.375-0.46)
Wyoming	0.32 (0.207-0.473)

Table S6. Cardiovascular Disease and Sarcoidosis-Related Age-Adjusted Mortality Rates per 100,000, Stratified by States in the United States, 2021 to 2022

State	Age-Adjusted Rate (95%CI)
Alabama	0.681 (0.51-0.891)
Alaska	Suppressed (Suppressed-Suppressed)
Arizona	0.284 (0.189-0.411)
Arkansas	Unreliable (0.227-0.644)
California	0.354 (0.303-0.404)
Colorado	0.742 (0.562-0.961)
Connecticut	0.779 (0.566-1.046)
Delaware	Unreliable (0.499-1.418)
District of Columbia	3.804 (2.585-5.399)
Florida	0.405 (0.342-0.468)
Georgia	0.724 (0.59-0.857)
Hawaii	Suppressed (Suppressed-Suppressed)
Idaho	Unreliable (0.288-0.848)
Illinois	0.54 (0.435-0.644)
Indiana	0.511 (0.381-0.672)
Iowa	0.484 (0.319-0.704)
Kansas	0.499 (0.317-0.749)
Kentucky	0.644 (0.468-0.864)
Louisiana	0.613 (0.441-0.828)
Maine	Unreliable (0.447-1.269)
Maryland	1.808 (1.546-2.07)
Massachusetts	0.514 (0.384-0.674)
Michigan	0.64 (0.52-0.76)
Minnesota	0.947 (0.749-1.182)
Mississippi	1.071 (0.775-1.442)
Missouri	0.392 (0.275-0.543)
Montana	Suppressed (Suppressed-Suppressed)
Nebraska	Unreliable (0.292-0.861)
Nevada	0.477 (0.308-0.704)
New Hampshire	Unreliable (0.299-0.96)
New Jersey	0.555 (0.439-0.693)
New Mexico	Suppressed (Suppressed-Suppressed)
New York	0.647 (0.559-0.735)
North Carolina	0.974 (0.82-1.129)
North Dakota	Suppressed (Suppressed-Suppressed)
Ohio	0.773 (0.646-0.901)
Oklahoma	0.673 (0.471-0.932)
Oregon	0.566 (0.406-0.768)
Pennsylvania	0.773 (0.657-0.889)
Rhode Island	Unreliable (0.354-1.269)
South Carolina	1.281 (1.035-1.527)
South Dakota	Suppressed (Suppressed-Suppressed)
Tennessee	0.538 (0.411-0.693)
Texas	0.444 (0.378-0.51)

Utah	0.556 (0.34-0.859)
Vermont	Unreliable (0.618-1.896)
Virginia	0.777 (0.625-0.929)
Washington	0.898 (0.723-1.074)
West Virginia	0.798 (0.511-1.188)
Wisconsin	0.864 (0.685-1.075)
Wyoming	Suppressed (Suppressed-Suppressed)

Table S7. Cardiovascular Disease and Sarcoidosis-Related Age-Adjusted Mortality Rates per 100,000, Stratified by Census Region in the United States, 1999 to 2022

Census Region	Year	Age-Adjusted Rate (95% CI)
**Northeast**	1999	0.368 (0.306-0.431)
**Northeast**	2000	0.4 (0.335-0.464)
**Northeast**	2001	0.493 (0.42-0.566)
**Northeast**	2002	0.505 (0.433-0.577)
**Northeast**	2003	0.424 (0.357-0.49)
**Northeast**	2004	0.45 (0.382-0.518)
**Northeast**	2005	0.445 (0.376-0.515)
**Northeast**	2006	0.464 (0.395-0.533)
**Northeast**	2007	0.45 (0.382-0.518)
**Northeast**	2008	0.43 (0.364-0.496)
**Northeast**	2009	0.448 (0.382-0.513)
**Northeast**	2010	0.478 (0.411-0.544)
**Northeast**	2011	0.485 (0.414-0.555)
**Northeast**	2012	0.462 (0.394-0.529)
**Northeast**	2013	0.443 (0.38-0.506)
**Northeast**	2014	0.46 (0.394-0.526)
**Northeast**	2015	0.502 (0.435-0.568)
**Northeast**	2016	0.482 (0.417-0.547)
**Northeast**	2017	0.448 (0.385-0.511)
**Northeast**	2018	0.494 (0.43-0.559)
**Northeast**	2019	0.493 (0.429-0.558)
**Northeast**	2020	0.64 (0.566-0.714)
**Northeast**	2021	0.697 (0.622-0.771)
**Northeast**	2022	0.593 (0.524-0.662)
**Midwest**	1999	0.242 (0.196-0.288)
**Midwest**	2000	0.332 (0.277-0.387)
**Midwest**	2001	0.369 (0.31-0.428)
**Midwest**	2002	0.311 (0.258-0.363)
**Midwest**	2003	0.369 (0.313-0.425)
**Midwest**	2004	0.371 (0.313-0.429)
**Midwest**	2005	0.34 (0.286-0.394)
**Midwest**	2006	0.398 (0.339-0.457)
**Midwest**	2007	0.368 (0.313-0.423)
**Midwest**	2008	0.381 (0.323-0.438)
**Midwest**	2009	0.369 (0.313-0.425)

**Midwest**	2010	0.43 (0.369-0.49)
**Midwest**	2011	0.42 (0.362-0.479)
**Midwest**	2012	0.364 (0.307-0.421)
**Midwest**	2013	0.453 (0.394-0.512)
**Midwest**	2014	0.434 (0.376-0.492)
**Midwest**	2015	0.449 (0.389-0.509)
**Midwest**	2016	0.422 (0.366-0.478)
**Midwest**	2017	0.492 (0.432-0.553)
**Midwest**	2018	0.48 (0.421-0.538)
**Midwest**	2019	0.481 (0.421-0.542)
**Midwest**	2020	0.597 (0.531-0.662)
**Midwest**	2021	0.645 (0.577-0.714)
**Midwest**	2022	0.604 (0.54-0.669)
**South**	1999	0.345 (0.299-0.391)
**South**	2000	0.448 (0.395-0.5)
**South**	2001	0.436 (0.384-0.487)
**South**	2002	0.41 (0.362-0.459)
**South**	2003	0.452 (0.4-0.504)
**South**	2004	0.464 (0.412-0.516)
**South**	2005	0.424 (0.375-0.473)
**South**	2006	0.463 (0.412-0.514)
**South**	2007	0.393 (0.348-0.438)
**South**	2008	0.479 (0.43-0.528)
**South**	2009	0.522 (0.47-0.574)
**South**	2010	0.459 (0.412-0.505)
**South**	2011	0.517 (0.466-0.568)
**South**	2012	0.478 (0.43-0.527)
**South**	2013	0.469 (0.423-0.515)
**South**	2014	0.498 (0.451-0.546)
**South**	2015	0.517 (0.469-0.564)
**South**	2016	0.534 (0.484-0.584)
**South**	2017	0.488 (0.441-0.534)
**South**	2018	0.54 (0.492-0.587)
**South**	2019	0.59 (0.54-0.641)
**South**	2020	0.656 (0.604-0.708)
**South**	2021	0.715 (0.661-0.769)
**South**	2022	0.697 (0.644-0.749)
**West**	1999	0.157 (0.118-0.205)
**West**	2000	0.185 (0.144-0.234)
**West**	2001	0.222 (0.178-0.275)
**West**	2002	0.26 (0.21-0.319)
**West**	2003	0.281 (0.228-0.334)
**West**	2004	0.297 (0.243-0.351)
**West**	2005	0.266 (0.216-0.316)
**West**	2006	0.281 (0.231-0.332)
**West**	2007	0.298 (0.246-0.351)
**West**	2008	0.26 (0.211-0.31)
**West**	2009	0.309 (0.258-0.36)
**West**	2010	0.228 (0.185-0.271)

**West**	2011	0.296 (0.247-0.346)
**West**	2012	0.255 (0.211-0.298)
**West**	2013	0.28 (0.231-0.328)
**West**	2014	0.296 (0.247-0.345)
**West**	2015	0.287 (0.24-0.334)
**West**	2016	0.348 (0.297-0.4)
**West**	2017	0.376 (0.324-0.429)
**West**	2018	0.369 (0.318-0.421)
**West**	2019	0.379 (0.328-0.431)
**West**	2020	0.405 (0.353-0.457)
**West**	2021	0.461 (0.404-0.518)
**West**	2022	0.441 (0.387-0.496)

Table S8. Cardiovascular Disease and Sarcoidosis Related Age-Adjusted Mortality Rates per 100,000 in United States stratified by Urban-Rural Classification, 1999-2020

	**Age-Adjusted Rate (95% CI)**	
**Year**	**Urban**	**Rural**
1999	0.29 (0.262-0.317)	0.259 (0.203-0.326)
2000	0.378 (0.347-0.41)	0.294 (0.234-0.365)
2001	0.383 (0.351-0.414)	0.325 (0.261-0.389)
2002	0.379 (0.349-0.41)	0.298 (0.239-0.367)
2003	0.396 (0.364-0.428)	0.323 (0.261-0.385)
2004	0.38 (0.35-0.409)	0.305 (0.246-0.364)
2005	0.363 (0.334-0.392)	0.309 (0.249-0.368)
2006	0.422 (0.391-0.454)	0.308 (0.25-0.366)
2007	0.392 (0.362-0.422)	0.307 (0.244-0.369)
2008	0.429 (0.397-0.461)	0.34 (0.278-0.402)
2009	0.425 (0.395-0.456)	0.374 (0.31-0.438)
2010	0.417 (0.387-0.447)	0.399 (0.332-0.466)
2011	0.441 (0.41-0.473)	0.376 (0.309-0.443)
2012	0.391 (0.362-0.419)	0.383 (0.317-0.45)
2013	0.402 (0.374-0.43)	0.423 (0.356-0.491)
2014	0.42 (0.392-0.449)	0.446 (0.373-0.518)
2015	0.476 (0.444-0.508)	0.39 (0.325-0.455)
2016	0.45 (0.42-0.479)	0.407 (0.339-0.474)
2017	0.474 (0.443-0.504)	0.434 (0.361-0.507)
2018	0.476 (0.447-0.505)	0.425 (0.357-0.492)
2019	0.491 (0.461-0.521)	0.471 (0.4-0.541)
2020	0.578 (0.546-0.61)	0.592 (0.516-0.668)

Table S9. Cardiovascular Disease and Sarcoidosis-Related Age-Adjusted Mortality Rates (AAMR) per 100,000 individuals Stratified by Ten Year Age Groups in the United States, 1999 to 2022

	Crude Mortality Rates (95% CI)		
**Year**	**25-44 Year**	**45-64 Year**	**65 and above**
1999	0.109 (0.086-0.133)	0.379 (0.329-0.428)	0.571 (0.49-0.652)
2000	0.155 (0.126-0.183)	0.5 (0.445-0.555)	0.583 (0.505-0.661)
2001	0.109 (0.087-0.131)	0.539 (0.482-0.597)	0.7 (0.613-0.787)
2002	0.109 (0.088-0.13)	0.479 (0.427-0.53)	0.748 (0.656-0.839)
2003	0.164 (0.133-0.194)	0.479 (0.426-0.531)	0.735 (0.649-0.822)
2004	0.109 (0.086-0.132)	0.518 (0.465-0.571)	0.788 (0.697-0.879)
2005	0.109 (0.085-0.133)	0.479 (0.429-0.529)	0.76 (0.67-0.85)
2006	0.109 (0.086-0.133)	0.479 (0.431-0.526)	0.823 (0.732-0.914)
2007	0.109 (0.085-0.138)	0.479 (0.429-0.528)	0.885 (0.79-0.979)
2008	0.109 (0.086-0.137)	0.479 (0.432-0.526)	0.868 (0.775-0.96)
2009	0.109 (0.086-0.132)	0.479 (0.433-0.524)	0.971 (0.874-1.068)
2010	0.155 (0.124-0.19)	0.479 (0.431-0.526)	1.048 (0.946-1.15)
2011	0.155 (0.124-0.191)	0.479 (0.431-0.526)	1.096 (0.995-1.196)
2012	0.109 (0.084-0.139)	0.479 (0.431-0.526)	0.954 (0.862-1.046)
2013	0.109 (0.084-0.14)	0.518 (0.47-0.566)	1.032 (0.937-1.128)
2014	0.055 (0.042-0.07)	0.518 (0.47-0.566)	1.031 (0.938-1.124)
2015	0.109 (0.085-0.138)	0.539 (0.488-0.59)	1.174 (1.073-1.275)
2016	0.055 (0.04-0.072)	0.518 (0.469-0.567)	1.214 (1.115-1.314)
2017	0.109 (0.084-0.14)	0.518 (0.469-0.566)	1.203 (1.106-1.301)
2018	0.055 (0.041-0.07)	0.518 (0.469-0.566)	1.328 (1.228-1.428)
2019	0.055 (0.041-0.071)	0.518 (0.469-0.566)	1.439 (1.335-1.543)
2020	0.055 (0.041-0.071)	0.596 (0.546-0.646)	1.671 (1.561-1.781)
2021	0.109 (0.086-0.137)	0.596 (0.546-0.647)	1.934 (1.816-2.053)
2022	0.055 (0.042-0.07)	0.596 (0.544-0.649)	1.863 (1.749-1.977)

Table S10. Sarcoidosis and COVID-19-related Age-Adjusted Mortality Rates per 100,000 in the United States from 2020 to 2022

Year	Deaths	Age-Adjusted Rate (95% CI)
2020	350	0.1245 (0.1109-0.1381)
2021	509	0.1665 (0.1513-0.1817)
2020	326	0.0973 (0.0863-0.1084)
